# Apolipoprotein-A1 as a Damage-Associated Molecular Patterns Protein in Osteoarthritis: *Ex Vivo* and *In Vitro* Pro-Inflammatory Properties

**DOI:** 10.1371/journal.pone.0122904

**Published:** 2015-04-07

**Authors:** Dominique de Seny, Gaël Cobraiville, Edith Charlier, Sophie Neuville, Laurence Lutteri, Caroline Le Goff, Denis Malaise, Olivier Malaise, Jean-Paul Chapelle, Biserka Relic, Michel G. Malaise

**Affiliations:** 1 Laboratory of Rheumatology, GIGA Research, University of Liege and CHU Hospital of Liege, 4000 Liège, Belgium; 2 Laboratory of Clinical Chemistry, CHU Hospital of Liege, 4000 Liège, Belgium; Katholieke Universiteit Leuven, BELGIUM

## Abstract

Osteoarthritis (OA) is associated with a local inflammatory process. Dyslipidemia is known to be an underlying cause for the development of OA. Therefore, lipid and inflammatory levels were quantified *ex vivo* in blood and synovial fluid of OA patients (n=29) and compared to those of rheumatoid arthritis (RA) patients (n=27) or healthy volunteers (HV) (n=35). The role of apolipoprotein A-I (ApoA1) was investigated *in vitro* on inflammatory parameters using human joint cells isolated from cartilage and synovial membrane obtained from OA patients after joint replacement. Cells were stimulated with ApoA1 in the presence or not of serum amyloid A (SAA) protein and/or lipoproteins (LDL and HDL) at physiological concentration observed in OA synovial fluid. In our *ex vivo* study, ApoA1, LDL-C and total cholesterol levels were strongly correlated to each other inside the OA joint cavity whereas same levels were not or weakly correlated to their corresponding serum levels. In OA synovial fluid, ApoA1 was not as strongly correlated to HDL as observed in OA serum or in RA synovial fluid, suggesting a dissociative level between ApoA1 and HDL in OA synovial fluid. *In vitro*, ApoA1 induced IL-6, MMP-1 and MMP-3 expression by primary chondrocytes and fibroblast-like synoviocytes through TLR4 receptor. HDL and LDL attenuated joint inflammatory response induced by ApoA1 and SAA in a ratio dependent manner. In conclusion, a dysregulated lipidic profile in the synovial fluid of OA patients was observed and was correlated with inflammatory parameters in the OA joint cavity. Pro-inflammatory properties of ApoA1 were confirmed *in vitro*.

## Introduction

Osteoarthritis (OA) is one of the most common chronic joint diseases causing substantial health deficits and becoming increasingly more prevalent as the population ages. Obesity is a major risk factor for developing OA and recent data suggest that there will be soon an epidemic of obesity-related osteoarthritis in the general population [1].

OA is characterized by a dysregulation of the normal joint homeostasis that leads to intra-articular cartilage degradation, attempted repair and a local inflammatory process. Articular cartilage is a specific connective tissue covering joint surfaces. It has a glistening, white appearance. Microscopically, it is composed of water, collagen, proteoglycan and a wide range of matrix proteins and lipids. Chondrocyte is a unique cell type in articular cartilage tissue and is essential for producing a large amount of extracellular matrix. The synovial membrane is a layer of connective tissue that covers joint cavities and makes lubrication of articular cartilage. It is composed of fibroblast-like synoviocytes. In OA, catabolic hyperactivity of chondrocytes is observed. The synovial membrane and chondrocytes start to secrete inflammatory mediators (cytokines and metallo-proteinases) enhancing extracellular matrix degradation in cartilage. Furthermore, anabolic activities of chondrocytes that regulate the synthesis of extracellular matrix components and maintain the functional integrity of joints are decreased, thereby impeding any cartilage repair. OA was recently considered as part of the metabolic syndrome rather than only due to aging or mechanical stress [2]. Several evidences point to the direction of an altered lipid metabolism as an underlying contributing factor for the development of OA. First, it is a systemic disorder in which homeostatic dysregulation within the joint structure might be due to adipokines activities [3]. Second, many researches have demonstrated that overweight people [4], people with diabetes [5] or people with elevated serum cholesterol [6] had a much greater risk to develop OA compared to healthy persons. Third, excess fat mass, particularly central or visceral adipose tissue enhances insulin resistance, hyperinsulinemia, glucose intolerance, hypertension, dyslipidemia and cardiovascular disorders, symptoms that can be linked to OA [7, 8]. Fourth, lipid deposition in the joint is observed at the early stages of OA [9]. Finally, several similarities between OA and atherosclerosis in their underlying aetiopathogenic factors have suggested chondrocytes as potential foam cells in OA [10].

Recently, we have studied the role played by an apolipoprotein, the acute phase serum amyloid A (A-SAA), as a pro-inflammatory marker in OA joints [11]. Under non-inflammatory conditions, A-SAA protein is associated with high-density lipoprotein (HDL) in plasma [12]. Liver predominantly produces A-SAA, and its concentration in blood may increase up to 1000 fold during the acute phase of inflammation. A-SAA levels in serum of OA patients are significantly higher to those in corresponding synovial fluid, but both levels are closely correlated to each other. A-SAA serum levels are also correlated with the Kellgren & Lauwrence score, a radiological score of severity in OA [11]. We therefore concluded that A-SAA in the synovial fluid of OA, but also of rheumatoid arthritis (RA) patients, was mainly due to an increased level of A-SAA in blood and probably due to the diffusion process into the joint cavity even though fibroblast-like synoviocytes (FLS) and chondrocytes can also contribute to a local production [11]. Increased level of A-SAA in blood or synovial fluid can significantly affect interactions between HDL and apolipoprotein A-I (ApoA1) and therefore affect properties of these lipoproteins [13, 14]. Indeed, ApoA1 is a major protein component of HDL cargo molecules in plasma and both are known to play a central role in the back transport of cholesterol from peripheral tissues to the liver.

In this study, ApoA1 levels and other lipidic/inflammatory parameters were quantified in synovial fluid and blood samples of OA patients, and were compared to those of RA patients (for blood and synovial fluid) and of healthy volunteers (HV) (for blood only). We also investigated the role played by ApoA1 compared to A-SAA on inflammatory parameters using human joint cells, primary chondrocytes and FLS, provided from OA patients. Because HDL is known to exert anti-inflammatory effects against a wide range of inflammatory agents, we sought to investigate whether HDL attenuates joint inflammatory responses in the presence of apolipoproteins such as ApoA1 and A-SAA.

## Materials and Methods

### Patients

Twenty-nine patients with OA and 27 with RA recruited through hospital outpatient clinics took part in this study. All patients fulfilled established diagnostic criteria for their respective diseases [15, 16]. None of the OA patients were on intra-articular steroid. Among the 27 RA patients, 11 (40%) received concomitant methotrexate at a mean dosage of 9.4 mg/week (range 6–15) and 15 (55%) prednisolone at a mean dosage of 8.9 mg/day (range 5–20). Thirty-five healthy volunteers (HV), matched for age [median (range) yr: 56 (50–64)], gender (56% of female) and BMI [median (range) Kg/m^2^: 25 (19–31)] served as the control group (Table 1). HV were qualified for entry into the study considering the following exclusion criteria: any prior history of knee trauma, joint pain, chronic inflammatory or autoimmune disease, cardiovascular disease, diabetes, corticosteroid injection or NSAID use. Subjects on lipid lowering agents or impaired hepatic function were also excluded. OA and RA patients were matched for the BMI of HV. None had been taking statins in the last 3 months of sample collection.

**Table 1:** Demographical characteristics.

	HV	OA	RA
Number of subjects	35	29	27
Female (%)	57	55	70
Age, median (range)	56 (50–64)	67 (53–86)	57 (19–73)
BMI, median (range) Kg/m^2^	25 (19–31)	27 (18–32)	25 (19–30)
MTX (%) [Mean dosage (range)]	0	0	40 [9.4 mg/week (6–15)]
Prednisolone (%)	0	0	55 [8.9 mg/week (5–20)]
[Mean dosage (range)]			
ApoA1 (g/L)—SF	/	0.52 (0.38–0.91)	0.80 (0.57–1.20)
ApoA1 (g/L)—Serum	1.64 (1.27–2.47)	1.65 (1.14–2.50)	1.57 (0.96–2.3)
A-SAA (μg/mL)—SF	/	3.5 (0.7–17)	31.1 (0.5–1832)
A-SAA (μg/mL)—Plasma	19 (5–88)	38 (6–123)	333 (13–4651)
HDL-C (g/L)—SF	/	0.26 (0.17–0.44)	0.32 (0.22–0.55)
HDL-C (g/L)—Serum	0.62 (0.34–1.03)	0.49 (0.28–1.00)	0.54 (0.28–0.9)
LDL-C (g/L)—SF	/	0.43 (0.06–0.98)	0.52 (0.26–1.26)
LDL-C (g/L)—Serum	1.23 (0.62–2.42)	1.46 (0.75–2.26)	1.23 (0.74–1.98)
anti—Ox LDL Ab (UI/L)—SF	/	76 (37–406)	126 (37–1200)
anti—Ox LDL Ab (UI/L)—Serum	171 (37–1139)	113 (37–929)	213 (37–1200)
Total Cholesterol (g/L)—SF	/	0.70 (0.28–1.42)	0.91 (0.48–1.65)
Total Cholesterol (g/L)—Serum	2.19 (1.58–3.03)	2.30 (1.43–2.95)	2.04 (1.32–2.74)
IL-6 (ng/mL)—SF	/	0.12 (0.01–4.09)	6.2 (0.04–125.2)
IL-6 (ng/mL)—Serum	<0.009	<0.009	0.03 (0.0015–0.183)
MMP-1 (ng/mL)—SF	/	49 (5–1115)	553 (7–4104)
MMP-1 (ng/mL)—Serum	1.34 (0.62–7.15)	1.14 (0.41–4.04)	3.53 (0.88–23.23)
MMP-3 (ng/mL)—SF	/	1292 (224–19238)	25144 (493–106173)
MMP-3 (ng/mL)—Serum	10.71 (5.79–37.87)	16 (5.9–63.18)	75 (19.08–293.63)

Apolipoprotein A-I (ApoA1), serum amyloid A (A-SAA), lipoproteins (LDL, HDL), anti-oxidized LDL antibodies, total cholesterol levels and inflammatory parameters in synovial fluids (SF) and serum (or plasma) of osteoarthritis (OA) and rheumatoid arthritis (RA) patients compared to those in matched healthy volunteers (HV). Values are median (range) otherwise indicated.

### Samples

Blood samples were collected from HV after an overnight fast. Similarly, blood samples and synovial fluids were collected together from each OA and RA patients after an overnight fast. Blood samples were allowed to coagulate in plain glass tubes during 30 minutes. Serum, plasma (EDTA tubes) and synovial fluid were obtained by centrifugation at 3000 rpm for 10 min. Supernatants were aliquoted and immediately frozen at −80°C until required for experiments.

### Ethics Statement

The research was carried out according to The Code of Ethics of the World Medical Association (Declaration of Helsinki).

The institutional review boards (Research Ethics Committee) of the University Hospital, CHU de Liège, Belgium, approved both the study protocol and the use of verbal informed consent to allow research procedures on biologic samples or tissues collected in out- and in-patients during their passage or stay at the CHU de Liège, as explained in the institutional information booklet written by the hospital and provided to each patient. Clinicians gave to patients an oral statement about our research. This statement included basic elements of our informed consent. Then, clinicians informed patients about authorizations obtained from the local Research Ethics Committee to perform studies and showed documents to those who wanted to read it. Clinicians gave sufficient time to patients to consider whether or not they wanted to participate in the research. After allowing the potential subject sufficient time, clinicians answered to any additional questions patients might have. Then clinicians obtained patient’s verbal consent to participate in the research. Strict data management retained anonymity of participants.

### Lipoproteins, anti-oxidized LDL antibodies, total cholesterol and ApoA1 quantification

Total cholesterol, HDL-cholesterol (HDL-C) and LDL-cholesterol (LDL-C) were measured in serum on a Roche Modular P Chemistry Analyzer (Roche Diagnostics) using a cholesterol oxidase enzymatic method, a direct magnesium/dextran sulfate method and a homogeneous enzymatic colorimetric assay (Roche Diagnostics GmbH, Mannheim, Germany), respectively.

Serum levels of ApoA1 were determined by an immunonephelometric method on a BNII nephelometer (Dade Behring/Siemens) with specific antibodies (Siemens, Marburg, Germany). Anti-oxidized LDL antibodies (anti-Ox LDL Ab) were measured by a kit based on an enzyme immunoassay for the quantitative determination of human IgG autoantibodies against oxidized low density lipoprotein in serum (Biomedica Medizin GmbH,Vienna, Austria). This assay was realized on an ETIMAX 3000 (Diasorin, Italy).

### Human primary chondrocytes and FLS

Primary chondrocytes and FLS were isolated from human cartilage and synovial tissue, respectively, of OA patients after surgical joint resection as described in [17–19]. Informed consents were obtained and experiments approved by the ethics committee of our academic hospital (CHU, Liège, Belgium). Briefly, primary chondrocytes (2x10^5^ cells/0.5ml of DMEM medium containing 10% FBS) were seeded in 24-well plates (BD Biosciences, USA) in triplicate and stimulated after 5 days of culture. FLS (5x10^4^ cells/0.5ml of DMEM medium containing 10% FBS) were used between passages 2–6. After a defined culture time, cellular supernatants were collected for ELISA tests and cells were harvested for protein extraction.

### Culture reagents

Culture reagents were purchased as follows: purified human ApoA1, purified human high density lipoprotein (HDL), purified human low density lipoprotein (LDL) (human source, Millipore, CA, USA), recombinant human ApoA1 (rhApoA1, *E*. *Coli* source, #350–11, Peprotech, USA), recombinant human SAA (rhSAA, E. Coli source, #300–13, Peprotech, USA), prednisolone (Sigma-Aldrich, St Louis, MI, USA), IgG rabbit polyclonal control, IgG1 mouse monoclonal (Santa Cruz, CA, USA), anti-RAGE IgG rabbit polyclonal (Abcam, UK, #ab37647), anti-CLA1/SR-B1 (BD Biosciences, USA, #610883), lipoxin A4 (Cayman Chemical, Bioconnect, NL), TAK242 (CLI-095, InvivoGen, CA, USA), sulfo-N-succinimidyl oleate (SSO, Santa Cruz, CA, USA), and polymyxin B (Calbiochem Novabiochem Corp., CA, USA).

### ELISA

A commercially available sandwich enzyme-linked immunosorbent assay was used for IL-6, MMP-1 and MMP-3 (R&D Systems, Minneapolis, MN, USA), and A-SAA (Invitrogen, Belgium) quantification in serum or plasma (for A-SAA), in synovial fluid and in the cell supernatant according to manufacturer’s instructions.

### Endotoxin contamination

Lonza Company (Vervier, Belgium) quantified endotoxin level in ApoA1, HDL, LDL (human source, Millipore, CA, USA), rhSAA and rhApoA1 (*E*. *Coli* source, #300–13 and #350–11, Peprotech, USA) proteins. Endotoxin level was measured at 0.1 ng per μg of ApoA1, 0.02 pg per μg of HDL, 0.03 pg per μg of LDL, 2.5 pg per μg of rhSAA and 0.03 ng/μg of rhApoA1.

The highest endotoxin level was detected in ApoA1 (human source) at 0.1 ng/μg, which was equivalent to 3ng/mL (or 30 EU/mL) endotoxin level when 30 µg of ApoA1 (1μM) was supplied to 1 mL of *in vitro* culture medium. For *in vitro* experiments, polymyxin B (MW = 1.4 kDa) was used for complexing and inactivating endotoxin at a molar concentration equivalent to ApoA1 (MW = 28kDa): 1μM. Efficacy of polymyxin B at 1μM was tested with 10–1–0.1 ng/mL of LPS on fibroblast-like synoviocytes and chondrocytes. Polymyxin was first incubated with LPS, ApoA1, rhApoA1 or rhSAA during 45 min at 37°C before stimulation of *in vitro* cells.

### Statistical analysis

**Fig 1:**
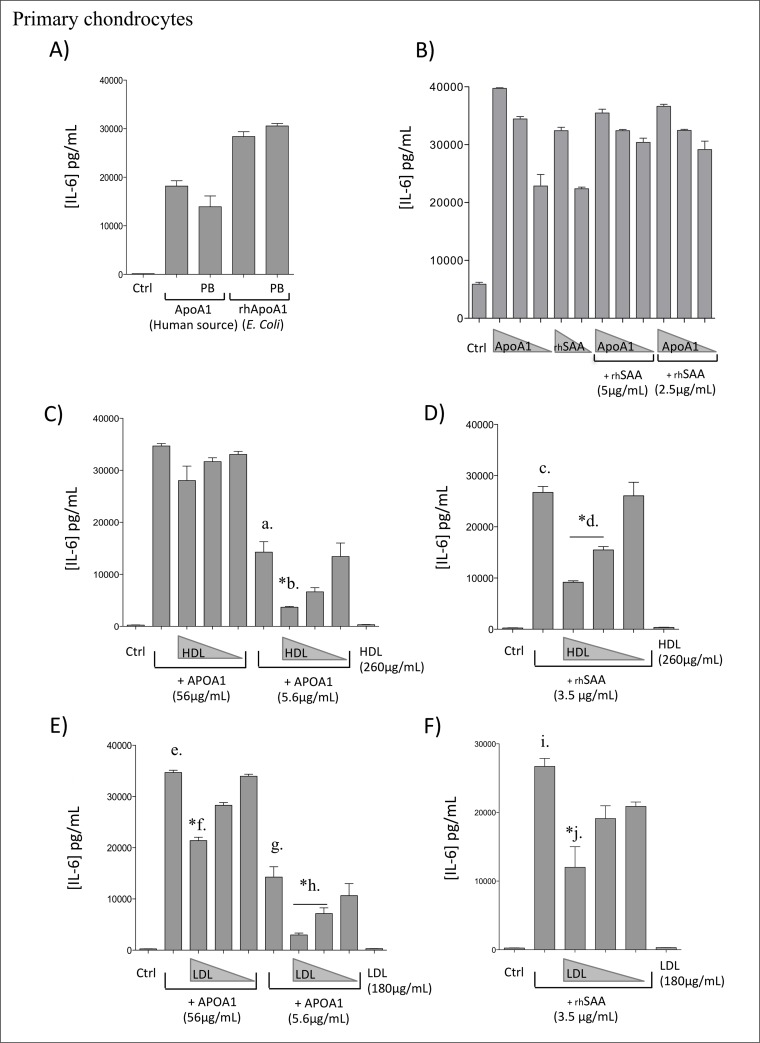
ApoA1-induced IL-6 expression after 24h using primary human chondrocytes. A) IL-6 expression after stimulation by ApoA1 (30μg/mL) in the presence or not of polymyxin B (PB) (1μg/mL). B) Dose response with ApoA1 (human source; 50-15-3μg/mL) and rhSAA (5–2.5μg/mL). C) ApoA1 (human source; 56–5.6μg/mL) with or without HDL (260-50-3μg/mL). D) rhSAA (3.5μg/mL) with or without HDL (260-50-3μg/mL). E) ApoA1 (human source; 56–5.6μg/mL) with or without LDL (180-30-2μg/mL). D) rhSAA (3.5μg/mL) with or without LDL (180-30-2μg/mL). *b,*d,*f,*h and *j statistically different to “a”, “c”, “e”, “g” and “i”, respectively. Data were expressed as means ± SEM (n = 3). * P< 0.05 by Mann–Whitney test.

**Fig 2:**
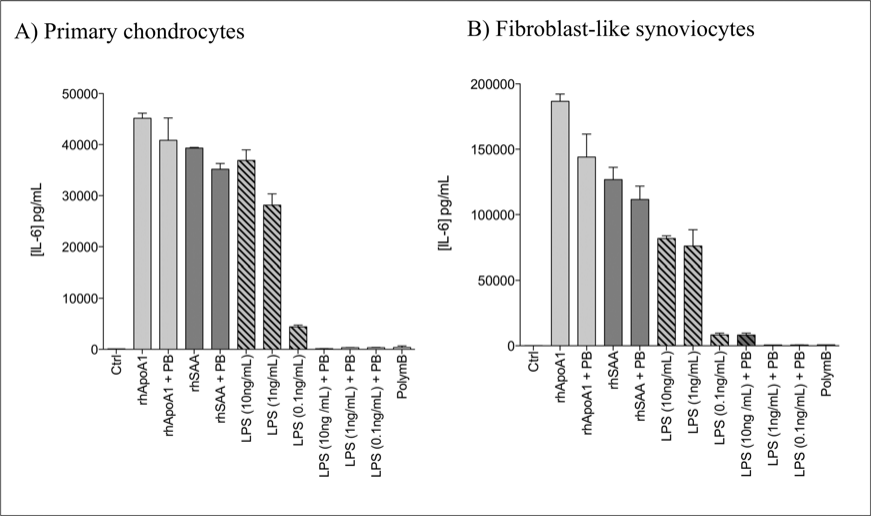
Endotoxin contamination and polymyxin B efficacy using primary human chondrocytes and fibroblast-like synoviocytes (FLS). A) Primary human chondrocytes and B) FLS were stimulated with rhApoA1 (30μg/mL) or rhSAA (3.5μg/mL) or LPS (10-1-0.1 μg/mL) in the presence or not of polymyxin B (1μg/mL).

**Fig 3:**
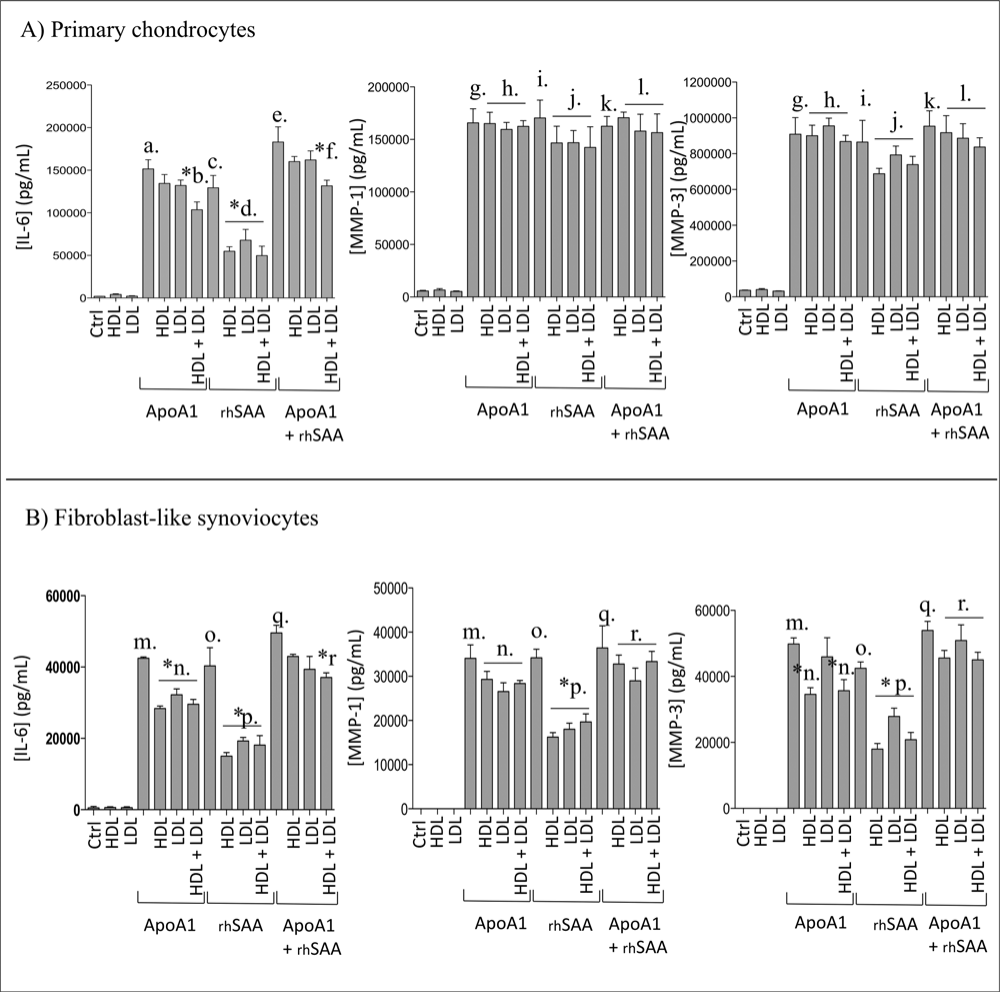
ApoA1-induced IL6, MMP-1 and MMP-3 expressions in the presence of lipoproteins after 24h using primary human chondrocytes and fibroblast-like synoviocytes (FLS). A) Primary human chondrocytes and B) FLS were stimulated with ApoA1 (human source) (56μg/mL), rhSAA (3.5μg/mL), HDL (260μg/mL) and LDL (180μg/mL). Data were expressed as means ± SEM (n = 3). * P< 0.05 by Mann–Whitney test.

**Fig 4:**
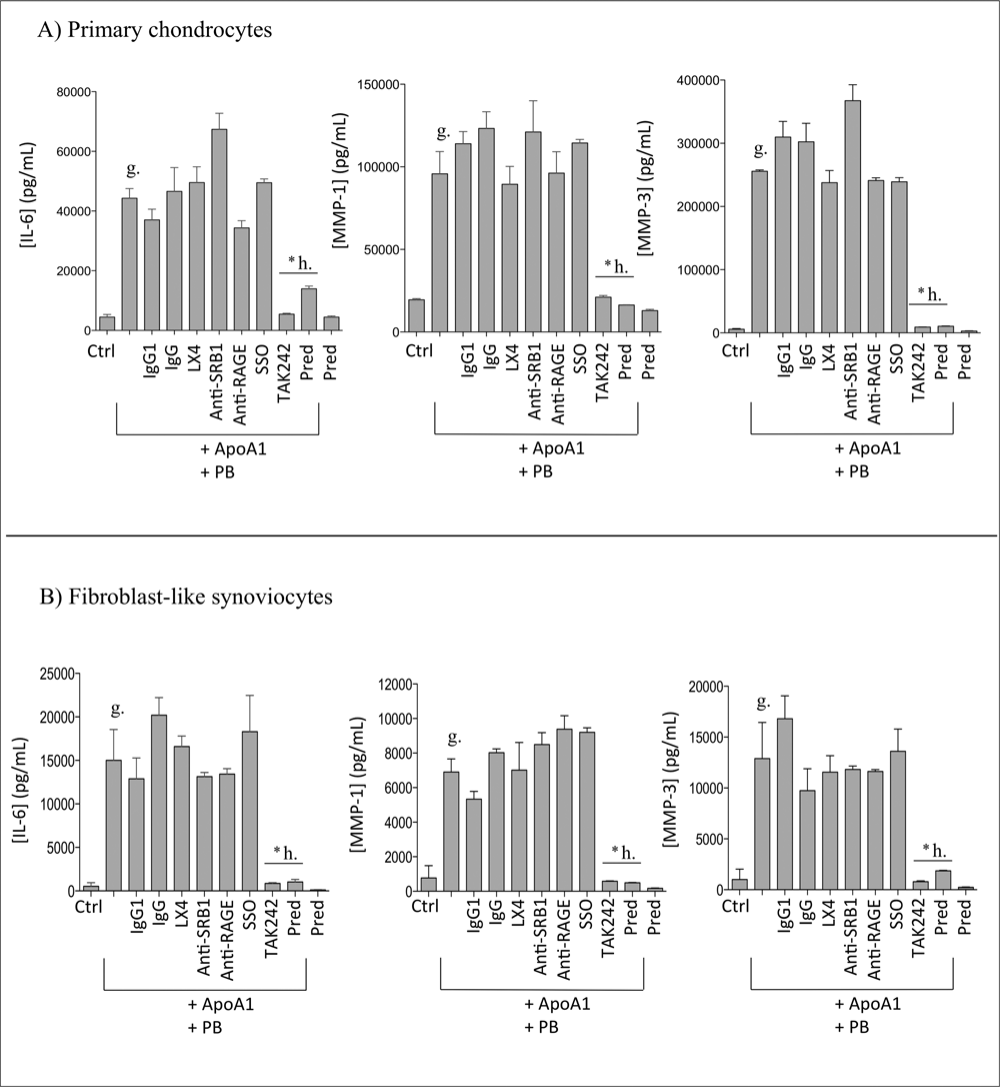
TAK242 reduced ApoA1-induced IL-6, MMP-1 and MMP-3 expressions in human primary chondrocytes and fibroblast-like synoviocytes (FLS). A) Primary human Chondrocytes and B) FLS were stimulated with ApoA1 (30μg/mL) in the presence or not of several inhibitors: lipoxin A4 (LX4; 1μM), anti-SR-B1 antibody (5μg/mL) and its isotype IgG1 control antibody (5μg/mL), anti-RAGE (10μg/mL) and its isotype IgG control antibody (10μg/mL) and TAK242 (1μM) for FPRL1, SR-B1, RAGE and TLR4 receptors, respectively. Polymyxin B was used at a concentration of 1μg/mL. *b and *h statistically different to “a” and “g”, respectively. Data were expressed as the means ± SEM (n = 3). * P< 0.05 by Mann–Whitney test.

Values in Table 1 are median (range) otherwise indicated. Correlation coefficients were obtained by the Spearman rank correlation test in Tables 2 and 3. Statistical analysis was performed by GraphPad Prism software version 4.01 for Windows. Graphs in Figs 1, 2, 3 and 4 represent means of triplicates from three independent experiments (n = 3); error bars indicate standard errors of the means (SEM). P-values were obtained using the Mann-Whitney U-test. A P-value < 0.05 was considered as statistically significant.

**Table 2:** Synovial fluid *vs*. blood levels.

OA samples	r	P-value	
ApoA1	0.43	0.035	*
LDL-C	0.44	0.025	*
Tot. Cholesterol	0.40	0.045	*
HDL-C	0.68	0.0001	***
A-SAA	0.86	P<0.0001	***
anti-OxLDL Ab	0.80	P<0.0001	***
IL-6	NA	NA	NA
MMP-1	0.37	0.046	*
MMP-3	0.40	0.04	*
RA samples			
ApoA1	0.56	0.020	*
LDL-C	0.33	0.14	NS
Tot. Cholesterol	0.08	0.71	NS
HDL-C	0.63	0.0016	**
A-SAA	0.94	P<0.0001	***
anti-OxLDL Ab	0.71	P<0.0001	***
IL-6	0.51	0.009	**
MMP-1	0.14	0.052	NS
MMP-3	0.57	0.0031	**

Correlations between synovial fluid and serum lipoproteins, anti-oxidized LDL antibodies, total cholesterol, apolipoprotein A-I and inflammatory parameters in the whole group of osteoarthritis (OA) or rheumatoid arthritis (RA) patients. Correlation coefficients (r) were obtained by the Spearman rank correlation test. NS; non-significant. NA; not applicable.

*P≤0.05

**P≤0.01

***P≤0.001

**Table 3:** Correlations between parameters detected in serum and synovial fluid.

		HV				OA				RA			
		Serum		SF		Serum		SF		Serum		SF	
		r	P value	r	P value	r	P value	r	P value	r	P value	r	P value
ApoA1	*vs*. Tot. Cholest	0.12	0.5	NA	NA	0.11	0.56	0.72***	<0.0001	0.51**	0.01	0.33	0.14
ApoA1	*vs*. LDL-C	-0.34*	0.041	NA	NA	-0.08	0.68	**0.64** ***	<0.0001	0.11	0.64	-0.03	0.9
ApoA1	*vs*. HDL-C	0.94***	<0.0001	NA	NA	0.91***	<0.0001	**0.58** **	0.003	0.83***	<0.0001	0.79***	<0.0001
LDL-C	*vs*. HDL-C	-0.44**	0.008	NA	NA	-0.07	0.7	**0.4** *	0.04	0.11	0.64	0.21	0.3
Tot. Cholest.	*vs*. HDL-C	-0.02	0.92	NA	NA	0.09	0.63	**0.51** **	0.007	0.47**	0.01	0.47**	0.01
Tot. Cholest.	*vs*. LDL-C	0.76***	<0.0001	NA	NA	0.92***	<0.0001	**0.96** ***	<0.0001	**0.82** ***	<0.0001	**0.86** ***	<0.0001
ApoA1	*vs*. IL-6	NA	NA	NA	NA	NA	NA	**0.41** *	0.04	-0.26	0.23	0.21	0.35
ApoA1	*vs*. MMP-1	-0.23	0.18	NA	NA	-0.60***	<0.0001	0.22	0.3	-0.1	0.63	0.05	0.83
ApoA1	*vs*. MMP-3	-0.49**	0.003	NA	NA	-0.34*	0.047	0.34*	0.047	-0.21	0.32	-0.07	0.76
Tot. Cholest.	*vs*. IL-6	NA	NA	NA	NA	NA	NA	0.5**	0.008	0.16	0.47	0.4*	0.04
LDL-C	*vs*. IL-6	NA	NA	NA	NA	NA	NA	0.52**	0.006	0.28	0.19	0.27	0.18
HDL-C	*vs*. IL-6	NA	NA	NA	NA	NA	NA	0.08	0.7	-0.3	0.17	-0.08	0.7

Correlation coefficients (r) between levels of lipoproteins, apolipoproteins A-I, total cholesterol and IL-6. Correlation coefficients (r) were obtained by the Spearman rank correlation test.

*P≤0.05

**P≤0.01

***P≤0.001

HV = healthy volunteers; OA = osteoarthritis; RA = rheumatoid arthritis; SF = synovial fluid; NA = not applicable

## Results

### *Ex vivo* ApoA1 level in serum and synovial fluid of OA and RA patients

Blood samples were provided from healthy volunteers (n = 35), OA (n = 29) and RA (n = 27) patients, and synovial fluids were collected from OA and RA patients only. Synovial fluid from HV was in too small quantity to be taken safely and was therefore not collected. Demographical characteristics were summarized in Table 1. Age, BMI and gender of patients in the HV, OA and RA groups were not significantly different. ApoA1 level was measured in blood and synovial fluid and compared to other lipidic and inflammatory parameters.

### *Ex vivo*-ApoA1 status in OA synovial fluid

#### ApoA1 level inside the OA joint cavity is independently modulated from its corresponding serum level

Of interest, ApoA1, LDL-C and total cholesterol levels in synovial fluid were weakly correlated to their serum levels in OA [r = 0.43 (P = 0.035), 0.44 (P = 0.025) and 0.40 (P = 0.045), respectively] whereas strong correlations were observed for HDL-C (r = 0.68, P = 0.0001), A-SAA (r = 0.86, P<0.0001) and anti-OxLDL antibody (r = 0.80, P<0.0001) levels (Table 2). Similar results were observed in the RA group. These results suggest that ApoA1, LDL-C and total cholesterol levels in the OA and RA joint cavity are not only due to passive diffusion from blood to synovial fluid but are submitted to local modulation inside the cavity.

#### Correlations between ApoA1 levels and other lipidic and inflammatory parameters in OA synovial fluid

Unexpectedly, ApoA1 levels were highly correlated to total cholesterol (r = 0.72; P<0.0001) and LDL levels (r = 0.64, P<0.0001) inside the OA joint cavity but not inside their corresponding OA serum [r = 0.11 (P = 0.56); r = -0.08 (P = 0.68)] or inside RA synovial fluid [r = 0.33 (P = 0.14); r = -0.33 (P = 0.9)] (Table 3). Furthermore, correlation between ApoA1 and HDL inside the OA joint cavity was slightly decreased (r = 0.58, P<0.003) compared to its corresponding correlation inside OA serum (r = 0.91, P<0.0001). These data suggest that ApoA1 levels inside the OA joint cavity are altered; becoming significantly correlated to the total cholesterol and LDL levels but less significantly correlated to HDL levels. ApoA1 and LDL-C levels were also weakly but significantly correlated to IL-6 levels [r = 0.41 (P = 0.04); r = 0.52 (P = 0.006)], and ApoA1 to MMP-3 levels (r = 0.34, P = 0.047), another uncommon characteristic of OA synovial fluid suggesting a local dysregulated process within the lipidic and inflammatory parameters (Table 3).

### Pro-inflammatory properties of ApoA1 *in vitro*

ApoA1, A-SAA, HDL and LDL were estimated at a median level of 520 μg/mL, 3.5 μg/mL, 260 μg/mL and 430 μg/mL in OA synovial fluid (Table 1). Accordingly, similar concentrations were used, *in vitro*, to stimulate human primary chondrocytes and fibroblast-like synoviocytes, except for ApoA1 that was used at lower concentrations (56μg/mL or 30μg/mL), due to the too low initial commercialized ApoA1 concentration. Nevertheless, *in vitro* ApoA1 molar concentration (56 μg/mL, MW = 28kDa, 2μM) remained largely in excess compared to other molecules [HDL (260 μg/mL, MW = 200kDa, 1.3μM); LDL (180 μg/mL, MW = 2000kDa, 0.1μM) or to rhSAA (3.5 μg/mL, MW = 11.7kDa, 0.3μM)], as observed in OA pathophysiological conditions.

#### ApoA1 induced IL-6 expression in human primary chondrocytes

Human primary chondrocytes were stimulated with purified human ApoA1 (human source) or rhApoA1 (*E*.*Coli* source) proteins during 24h at a concentration of 30 μg/mL (Fig 1A). Both purified ApoA1 proteins (human and *E*. *Coli* source) induced IL-6 secretion by human primary chondrocytes in their respective cell medium (Fig 1A). Presence of polymyxin B (PB), a cationic cyclic lipopeptide that binds stoechiometrically the lipid A moiety of LPS/endotoxin and blocks its biological effects, did not significantly reduced ApoA1-induced IL-6 expression (Fig 1A). Polymyxin B efficacy is further described below.

In a previous work, we had already demonstrated that rhSAA induced cytokines and MMPs in a dose dependent manner [11]. In the following study, no synergic effect was observed when both ApoA1 (50–15–3 μg/mL) and rhSAA (5–2.5 μg/mL) proteins were combined for stimulation of human primary chondrocytes (Fig 1B).

We next analyzed whether HDL and LDL could themselves induce IL-6 secretion or modulate ApoA1-induced IL-6 expression. In Fig 1C, human primary chondrocytes were stimulated with ApoA1 56 μg/mL (2μM, excess of ApoA1) or 5.6 μg/mL (0.2μM, deficit of ApoA1) combined with decreasing HDL concentrations (260–50–3 μg/mL or 1.3–0.25–0.015μM). No HDL concentration could affect ApoA1-induced IL-6 expression when ApoA1 concentration was used in excess (56 μg/mL; 2 μM). However, HDL could significantly inhibit ApoA1-induced IL-6 expression when HDL (260 μg/mL, 1.3 μM) was highly in excess compared to ApoA1 (5.6 μg/mL, 0.2 μM) (a *vs*. *b., Fig 1C).

Human primary chondrocytes (Fig 1D) were then stimulated with rhSAA (3.5 μg/mL, 0.3 μM) and decreasing HDL concentrations (260–50–3 μg/mL or 1.3–0.25–0.015μM). RhSAA-induced IL-6 expression was reduced but only when HDL concentrations (260 μg/mL, 1.3 μM) were used in excess compared to rhSAA (c *vs*. *d, Fig 1D).

Similar experiments were performed using decreasing LDL concentrations (180–30–2 μg/mL or 0.1–0.015–0.001μM) (Fig 1E). LDL 180 μg/mL (0.1 μM) significantly reduced the ApoA1-induced IL-6 expression at both ApoA1 concentrations (56 μg/mL, 2 μM and 5.6 μg/ml, 0.2 μM) (e vs. *f; g vs. *h; Fig 1E), whereas lower LDL concentrations had no effect. RhSAA-induced IL-6 expressions were also significantly reduced when rhSAA 3.5 μg/ml (0.1 μM) was mixed with the higher concentration of LDL (180 μg, 0.1 μM), whereas lower concentrations had no effect (i vs *j, Fig 1F). Both HDL and LDL had no intrinsic capacities to stimulate IL-6 production by human primary chondrocytes, at least at concentrations tested (260 μg/mL for the HDL and 180 μg/mL for the LDL) (Fig 1C and 1F).

#### Endotoxin contamination and efficacy of polymyxin B

Efficacy of PB (1μg/mL) on endotoxin contamination was achieved using LPS (10–1–0.1 ng/mL) as a positive control on primary chondrocytes (Fig 2A) and fibroblast-like synoviocytes (Fig 2B). Results obtained were compared with ApoA1 (30μg/mL) and rhSAA (3.5μg/mL) (Fig 2A and 2B). Endotoxin contamination of ApoA1 and rhSAA in cell culture were estimated at 3ng/mL and 0.9ng/mL, respectively, according to Lonza (see Mat. and Meth. section) when 30μg of proteins were diluted in 1mL of culture medium. LPS (10ng/mL)—but not ApoA1- or rhSAA-induced IL-6 expression was blocked when LPS was combined with PB (1μg/mL) (Fig 2A and 2B). These results suggest that ApoA1 and rhSAA, but not LPS, was responsible for pro-inflammatory effects observed previously.

#### ApoA1 and rhSAA induced IL-6, MMP-1 and MMP-3 secretion by human primary chondrocytes and fibroblast-like synoviocytes in the presence of lipoproteins at OA pathophysiological level

We next analyzed the role of ApoA1, rhSAA, HDL, LDL and mixed conditions on IL-6, MMP-1 and MMP-3 expressions by human primary chondrocytes and by fibroblast-like synoviocytes. Selected concentrations were 56 μg/mL (2 μM) for ApoA1, 3.5 μg/mL (0.3 μM) for rhSAA, 260 μg/mL (1.3 μM) for HDL and 180 μg/mL (0.1 μM) for LDL.

For human primary chondrocytes (Fig 3A), we have observed that ApoA1- and rhSAA-induced IL-6 expression was significantly reduced but not abolished when rhSAA and/or ApoA1 were combined with [HDL and LDL] (a. *vs*. *b, c. *vs*. *d, e *vs*. *f). However, ApoA1- and rhSAA-induced MMP-1 and MMP-3 expressions remained unchanged in the presence of HDL and LDL (g. *vs*. h., i. *vs*. j. and k. *vs*. l.). For fibroblast-like synoviocytes (Fig 3B), we observed that rhSAA-induced IL-6, MMP-1 and MMP-3 expressions were significantly reduced but not abolished when rhSAA was combined with HDL and/or LDL (o. *vs*. *p). ApoA1-induced IL-6 and MMP-3 expressions were significantly reduced when ApoA1 was combined with HDL (m. *vs*. n.*). Finally, ApoA1- and rhSAA-induced IL-6 expression was significantly reduced when ApoA1 and rhSAA were combined with [HDL and LDL] (q *vs*. r*). Clearly, HDL, LDL or [HDL-LDL] inhibiting capacities on IL-6, MMP-1 and MMP-3 expressions induced by ApoA1 and A-SAA might be related to different molar ratios observed between ApoA1 or rhSAA and HDL or LDL.

#### TAK242 reduced ApoA1-induced proinflammatory signals in human primary chondrocytes and fibroblast-like synoviocytes

In our previous work, we demonstrated that cytokines and MMPs expression induced by rhSAA was mediated through toll-like receptor 4 (TLR4) [11]. In this work, we have also investigated several receptors including FPRL1, SR-B1/CLA1, CD36, TLR4 and RAGE. Several inhibitors were used to determine which receptor was involved in ApoA1-induced IL-6, MMP-1 and MMP-3 expression. LX4 is a competitor for FPRL1 binding. Anti-RAGE and anti-SR-B1 antibodies were used to block their respective receptors. They can be compared to their respective control: IgG and IgG1. SSO irreversibly inhibits CD36. In our *in vitro* system, none of these inhibitors could significantly inhibit ApoA1-induced IL-6, MMP-1 or MMP-3 expression (Fig 4A and 4B). However, TAK242, a small molecule known for binding selectively the intracellular domain of TLR4 among 10 different human TLRs [20] reduced drastically in both cell types ApoA1-induced pro-inflammatory signals similarly to the glucocorticoid, prednisolone (g. *vs*. *h, Fig 4A and 4B). Polymyxin B (1μg/mL) was used to avoid any LPS contamination.

## Discussion

Lipid diffusion into the joint cavity is dependent on the degree of inflammation [21]. Busso et *al*. have provided evidences that lipoprotein diffusion from the circulation into the synovial fluid was dependent on the disease type and particles size, and that the permeability of the barrier was increased under inflammatory conditions [22]. Apolipoproteins and lipoproteins seem therefore able to flow through the synovial barrier to enter into the joint cavity. Although inflammation is largely superior in RA compared to OA, we can nonetheless hypothesize that local inflammatory process and lipids diffusion inside OA joint cavity can also occur, as suggested by their detection in the synovial fluid of OA patients. Indeed, strong correlations have been raised between blood levels *versus* synovial levels for HDL, A-SAA and anti-OxLDL antibodies, suggesting that major damage of permeability can be discarded for both OA and RA synovial membrane. However, weak correlations have been raised for ApoA1, LDL-C and total cholesterol levels when blood levels were compared to synovial levels, enhancing the hypothesis of a local dysregulated process for these molecules inside the OA joint cavity. Interestingly, synovial fluid from OA patients displayed the unique characteristic of ApoA1 levels being strongly and positively correlated to LDL and cholesterol levels, which was not observed either in corresponding OA blood samples or RA synovial fluids. Conversely, ApoA1 was not as strongly correlated to HDL levels in OA synovial fluid compared to OA serum or HV and RA fluids, suggesting a dissociative level between ApoA1 and HDL inside the OA joint cavity. Finally, ApoA1, LDL-C and total cholesterol were also correlated to a local inflammatory process inside the OA joint cavity *via* IL-6 and MMP-3 parameters. Metalloproteinases are known for their activity on matrix cartilage degradation in OA. Oliviero *et al*. had already observed such correlation between ApoA1 and inflammatory markers *via* the white blood cell count parameter in the synovial fluid of OA patients [23].

### Inflammatory properties of ApoA1

ApoA1 is usually considered as an anti-inflammatory and atheroprotective agent [24]. However, it was recently demonstrated that high ApoA1 levels could predict the development of prehypertension [25] and type-2 diabetes [26]. Presence of ApoA1 was also observed in amyloid deposit of knee joint menisci and in acute gouty arthritis [27, 28]. The nature of ApoA1 as a covariate of inflammatory markers and a determinant in the metabolic syndrome (diabetes and prehypertension) was unexpected and further suggests its reconversion in an inflammatory apolipoprotein. Our *in vitro* studies confirmed the potential role of ApoA1 in inflammation by inducing a strong IL-6, MMP-1 and MMP-3 expression by primary chondrocytes and FLS. Increased protein expression levels of IL-8, MCP-1, GRO-α, TNF-α (for chondrocytes only) were also detected after 6h of ApoA1 stimulation in cells supernatants of human primary chondrocytes and FLS (data not shown). However, we could not detect any IL-1β protein secretion when cells were stimulated with ApoA1, in the limits of ELISA test (data not shown). Likewise A-SAA in our previous work [11], ApoA1-pro-inflammatory effects were mediated through the TLR4 receptor. Preliminary results indicated that NF-κB and MAPK downstream pathways were affected when cells were stimulated with ApoA1. Phosphorylation of IκBα and JNK were observed for both cell types whereas p38 phosphorylation was only observed for chondrocytes (data not shown). ApoA1 could therefore be considered as a potential “damage-associated molecular patterns (DAMPs)”. ApoA1 is known to play a role in the innate defense by binding Gram-negative bacteria [29] and having a high affinity for LPS. In our *in vitro* studies, LPS contamination was ruled out using polymyxin B. But, we cannot completely exclude that ApoA1/LPS complex decreased the threshold of pathogen-associated molecular patterns (PAMPs) recognition and in turn enhanced TLR4 signaling. Presence of bacterial peptidoglycan-polysaccharide complexes was already described in synovial tissues of patients with inflammatory OA [30]. These findings underline the necessity of clarifying whether ApoA1 itself binds to TLR4 or is a carrier of other particles mediating interaction with the receptor. Outer PAMP (such as LPS) could bind to extracellular DAMP (free ApoA1 particles) to form a complex that recognizes TLRs on synovial fibroblasts or chondrocytes, initiating a signaling cascade that leads to the secretion of inflammatory cytokines, production of tissue-destructive enzymes and finally resulting in OA. As described by Ospelt *et al*., TLR3 and TLR4 are among the most expressed receptor for TLRs1-10 in OA- and RA-synovial tissues, in normal synovial fibroblast and skin fibroblasts [31]. TLR4 expression was not significantly increased after ApoA1 stimulation of human chondrocytes or fibroblast-like synoviocytes (data not shown). But the dysregulated lipidic profile observed inside the OA joint cavity and most specifically, free ApoA1 particles could be involved, directly or indirectly, in the local inflammatory process by enhancing TLR4 activation. Onat *et al*. have also declared that ApoA1 could be converted into pro-inflammatory particles by aggregation to lipoprotein (a) Lp(a) [25]. They recently observed that low circulating Lp(a) associated with high ApoA1/HDL-C ratio were predicting factors for hypertriglyceridemic waist phenotype, which is considered as the core of the metabolic syndrome in impacting coronary heart disease risk [32]. ApoA1 could therefore become further diabetogenic, atherogenic [26, 33] and osteoarthritic.

### ApoA1 and lipoproteins

ApoA1 is the major structural protein of HDL, which plays a central role to shuttle cholesterol from peripheral tissues or cells to the liver [34]. Beside its participation in reverse cholesterol transport, HDL is associated to a large range of activities including anti-inflammatory, anti-pathogenic, anti-apoptotic and innate immune activities [35–38]. In our *in vitro* study, we have observed that HDL (as well as LDL) did not induce any pro-inflammatory mediators. Their action on ApoA1-induced IL-6 expression was dependent on both ApoA1 and HDL (or LDL) concentrations. Indeed, dose-ranging concentrations of HDL (or LDL) were unable to inhibit ApoA1-induced IL-6 expression when ApoA1 concentration was in excess (56 μg/ml), mimicking median OA synovial fluid levels. HDL was only able to inhibit IL-6 expression when ApoA1 concentration was ten-times lower (5.6μg/mL). Similarly, HDL (and LDL) actions on the rhSAA-induced IL-6 expression was also dependent on HDL (or LDL) concentrations. Pro-inflammatory properties of ApoA1 and A-SAA seemed therefore deeply influenced by local concentration of HDL or LDL and are [ApoA1/(HDL or LDL)] or [A-SAA/(HDL or LDL)] ratio dependent.

Recently, ApoA1 was also reported to interact with LDL (ApoA1-LDL) [39]. Interestingly, in this work, we have observed a high correlation between LDL and ApoA1 levels in OA synovial fluid but not in RA synovial fluid or in any serum samples group (HV, OA and RA).

Several studies have also demonstrated selective remodeling of HDL impairing its stability and functionality in inflammatory and pathological conditions [40]. Dyslipidemia, glycation and oxidative modifications on HDL were shown to be associated with impairment in HDL function [41].

Therefore, anti- and pro-inflammatory properties of lipoproteins (HDL and LDL) and apolipoproteins (ApoA1 and A-SAA) seem to be dependent on their pathophysiological environment. Our results suggest that a tight metabolic control of ApoA1 expression as well as HDL and LDL concentrations would influence the inflammatory properties of ApoA1 on chondrocytes and fibroblast-like synoviocytes, cells involved in the development of OA. Our results strengthen the concept that OA is also a metabolic syndrome, and not only due to aging or mechanical stress, in which homeostatic dysregulation within the joint structure might be due to apolipoproteins activities.
